# The role of integrin β1 in the heterogeneity of human embryonic stem cells culture

**DOI:** 10.1242/bio.034355

**Published:** 2018-11-15

**Authors:** Ade Kallas-Kivi, Annika Trei, Artjom Stepanjuk, Katrin Ruisu, Keiu Kask, Margus Pooga, Toivo Maimets

**Affiliations:** 1 Institute of Molecular and Cell Biology, University of Tartu, Riia 23, 51010 Tartu, Estonia; 2 Institute of Technology, University of Tartu, Nooruse 1, 50411 Tartu, Estonia

**Keywords:** Human embryonic stem cell, Pluripotency, Integrin β1, Differentiation, ECM, Embryoid body

## Abstract

The maintenance of the pluripotency of human embryonic stem (hES) cells requires special conditions for culturing. These conditions include specific growth factors containing media and extracellular matrix (ECM) or an appropriate substrate for adhesion. Interactions between the cells and ECM are mediated by integrins, which interact with the components of ECM in active conformation. This study focused on the characterisation of the role of integrin β1 in the adhesion, migration and differentiation of hES cells. Blocking integrin β1 abolished the adhesion of hES cells, decreasing their survival and pluripotency. This effect was in part rescued by the inhibition of RhoA signalling with Y-27632. The presence of Y-27632 increased the migration of hES cells and supported their differentiation into embryoid bodies. The differences in integrin β1 recycling in the phosphorylation of the myosin light chain and in the localisation of TSC2 were observed between the hES cells growing as a single-cell culture and in a colony. The hES cells at the centre and borders of the colony were found to have differences in their morphology, migration and signalling network activity. We concluded that the availability of integrin β1 was essential for the contraction, migration and differentiation ability of hES cells.

## Abstract (precis)

**Summary:** The interaction between integrin β1 and the extracellular matrix differs at the centre of the colony and at the periphery, and is crucial for the survival of embryonic stem cells.

## INTRODUCTION

Human embryonic stem (hES) cells require highly specific conditions for culturing, especially for large-scale production needed in translational therapy. For the efficient culturing of hES cells, pluripotency has to be maintained and spontaneous differentiation avoided. Thus, specific conditions need to be finely tuned, such as finding the proper substrate mimicking the extracellular matrix (ECM), the availability of growth factors and the utilisation of appropriate detachment and dissociation techniques. Under standardised conditions, hES cells grow in colonies. However, single-cell cultures are preferred under particular circumstances, such as in the case of the differentiation of cells towards a certain lineage. The maintenance of the pluripotency and prevention of the differentiation of hES cells has been a challenge and much effort has been invested into elaborating suitable cell culture media and establishing the best substrate for ECM attachment. Among these, the search for the best substrate to create xeno-free conditions for pluripotent hES cells has received special attention. However, the information about the interactions between hES cells and ECM substrates is still limited. Integrins are the leading players in hES cell–ECM interaction (Du et al., 2011) and different combinations of integrin β and α chains form heterodimers which recognise and bind a specific ligand. The integrin expression profile in hES cells has been shown to depend on the ECM substrate used for culturing. For instance, hES cells grown on laminin-511 fragment express a high amount of integrin α6β1 (Hongisto et al., 2012), while hES cells grown on Matrigel^®^-coated plates express various integrins (Vuoristo et al., 2009; Li et al., 2002). According to a recent observation, α6β1 is the most prominent integrin in hES cells and when the cells differentiate the expression of α6 integrin decreases (Vitillo et al., 2016). Integrins exist either in a bent or closed conformation that shows low affinity for ligands (inactive form) or in an extended or open state that shows high affinity for ligands (active form) (Gahmberg et al., 2009). Outside-in signalling is triggered by the ligand binding to the extracellular part of the integrin, whereas inside-out signalling is induced by the association of intracellular proteins, such as talin, to integrins which regulate their activity and promote the binding of the ligand. In addition, integrins are efficiently endocytosed during cell migration and upon stimulation and they switch between their active and inactive form (Arjonen et al., 2012; Shafaq-Zadah et al., 2016). The integrins could regulate different stages of hES cell–ECM interaction, for instance by establishing adhesion complexes and by influencing the migration of cells, thereby affecting the pluripotency and differentiation potential. Thus, the aforementioned properties and multi-layered functions make integrins a complicated object of study. Furthermore, an understanding of how ECM and its interactions with integrin receptors influence the pluripotency and differenctiation of ES cells is essential for developing protocols for efficient differentiation.

In this study, we focused on the role of integrin β1 in assembling focal adhesions and in regulating the contraction of hES cells that were grown in a colony or as a single-cell culture. The cross-talk of integrin β1 signalisation with RhoA and mTOR-controlled pathways was studied in pluripotent hES cells and in the cells differentiating into mesodermal lineage. The essentiality of integrin β1 for the survival and migration of pluripotent hES cells as well as for the formation of embryoid bodies was confirmed.

## RESULTS

### Localisation and expression of integrins β1 and α6 in hES cells

**Fig. 1.**
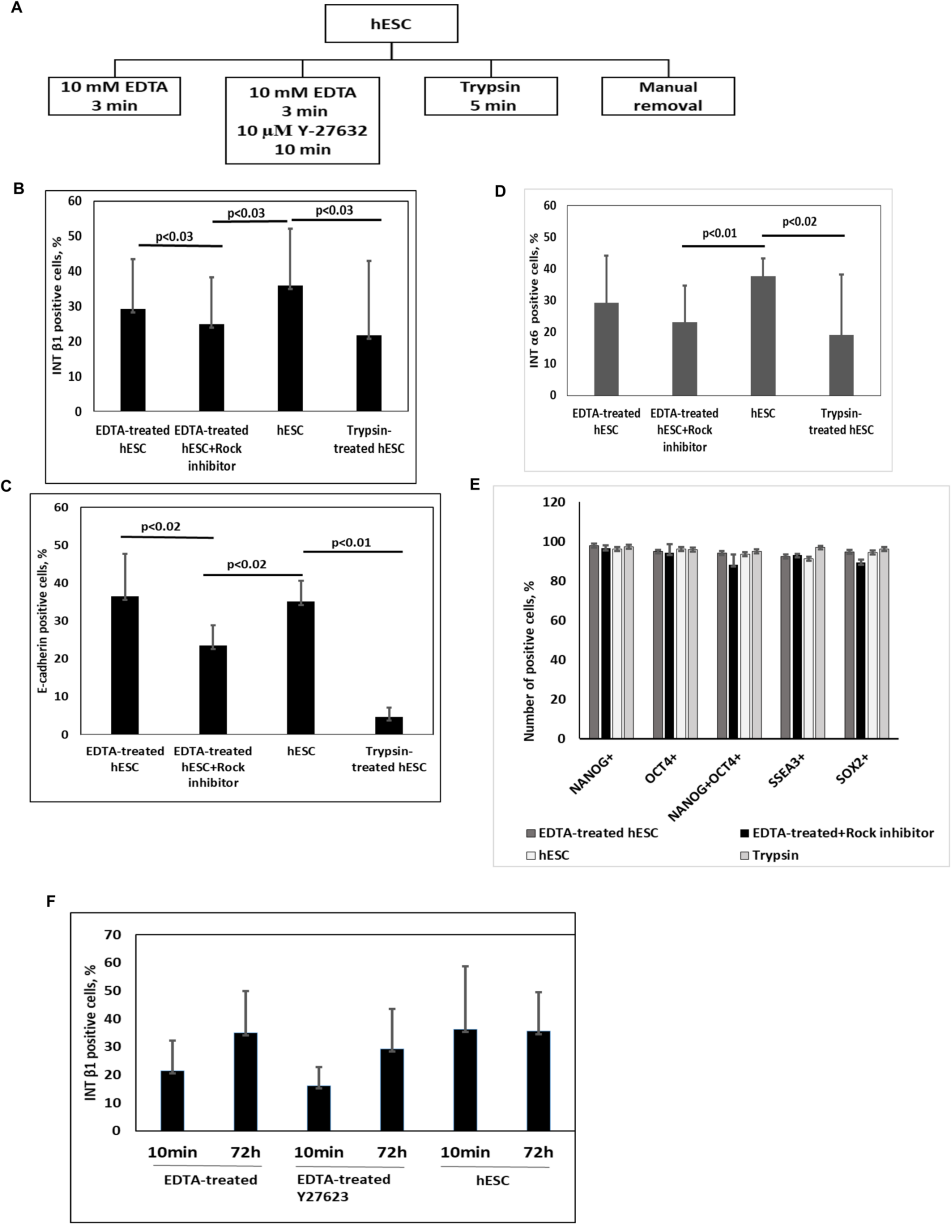
**The availability of integrin β1 on the plasma membrane of hES cells after detachment with different methods.** (A) The diagram shows the detachment methods for hES cells. The hES cells were dissociated and detached manually (hESC) or with EDTA with or without following the treatment with Y-27632 or with trypsin. (B) Comparison of integrin β1 levels on the plasma membrane of differently detached hES cells analysed by flow cytometry. The data were collected from seven independent experiments and are presented as mean±s.d. (C) Detection of E-cadherin on the plasma membrane of hES cells analysed by flow cytometry (*n*=4). (D) Comparison of integrin α6 levels on the plasma membrane of differently detached hES cells analysed by flow cytometry (*n*=5). (E) Comparison of the expression of pluripotency markers in differently detached hES cells analysed by flow cytometry. After the detachment of hES cells, the cells were fixed, permeabilised and stained with antibodies detecting the pluripotency markers NANOG, OCT4, SOX2 and SSEA3. The data have been collected from four independent experiments and are presented as mean±s.d. (F) Changes in the availability of integrin β1 on the plasma membrane of hES cell after 72 h analysed by flow cytometry. The data have been collected from four independent experiments and are presented as mean±s.d.

The adhesion of hES cells is triggered by the integrins available on the surface of the cell that interact with ECM components. First, we measured the levels of integrin β1 on hES cells by flow cytometry. In order to only detect integrin β1 on the membrane and to avoid the internalisation of the antigen-antibody complex, the hES cells were incubated with anti-integrin β1 antibodies on ice. We analysed the effect different agents used for the detachment of hES cells from the Matrigel^®^ matrix have on the levels of integrins on the plasma membrane (Fig. 1A). The treatment with trypsin reduced the levels of integrins β1 and α6 on the surface of hES cells significantly (Fig. 1B,D). Trypsin is known to drastically impair cell adhesion and is not suitable for re-seeding hES cells (Xu et al., 2010). The detachment of cells with ethylenediamine tetraacetic acid (EDTA) also decreased the levels of integrins β1 and α6 on the membrane, and ROCK inhibitor Y-27632 (treated for 10 min after detachment) further reduced the levels of integrins β1 and α6 (Fig. 1B,D). Since E-cadherin is reported to spatially control the adhesion of pluripotent stem cells (Toh et al., 2015), we analysed the levels of E-cadherin on the plasma membrane of hES cells and found that they were affected by the detachment of hES cells (Fig. 1C). In addition, western blot (WB) analysis confirmed the reduced levels of integrin β1, α6 and E-cadherin in trypsin-treated hES cells (Fig. S1). To assess whether the pluripotency of hES cells was affected by the reduced levels of integrins, we tested the expression of pluripotency markers NANOG, OCT4, SOX2 and SSEA3. However, no significant changes in the expression of pluripotency markers were detected by flow cytometry (Fig. 1E). This finding confirmed that the levels of integrin β1, α6 and E-cadherin on the plasma membrane could be affected during re-seeding, but such changes in the composition of the receptors on the plasma membrane did not influence the expression of the transcription factors responsible for pluripotency.

To find out whether the impaired availability of integrin β1 has any long-term consequences after re-seeding, the hES cells were detached using different methods and grown for a further 72 h. Flow cytometry revealed that the levels of integrin β1 on the membrane were similar in all groups notwithstanding the detachment techniques used for re-seeding, indicating that the initially induced differences on the level of adhesion molecules on the plasma membrane were lost during culturing and that the integrins were endocytosed and recycled back to the plasma membrane (Fig. 1F). We discovered that trypsin induced the degradation of integrin β1 on the plasma membrane and a substantial decrease in total integrin β1 protein levels, which might explain the lack of cell adhesion and survival. In addition, it has been previously reported that a decrease in E-cadherin is caused by trypsin treatment (Xu et al., 2010). It is important to note that after 72 h of growth, the single-cell culture (Y-27632 was included during the first 24 h) started to form colony-like structures (Fig. S2B) indicating that the surrounding microenvironment induced a targeted migration of cells to form cell-cell contacts and colonies round in shape.

**Fig. 2.**
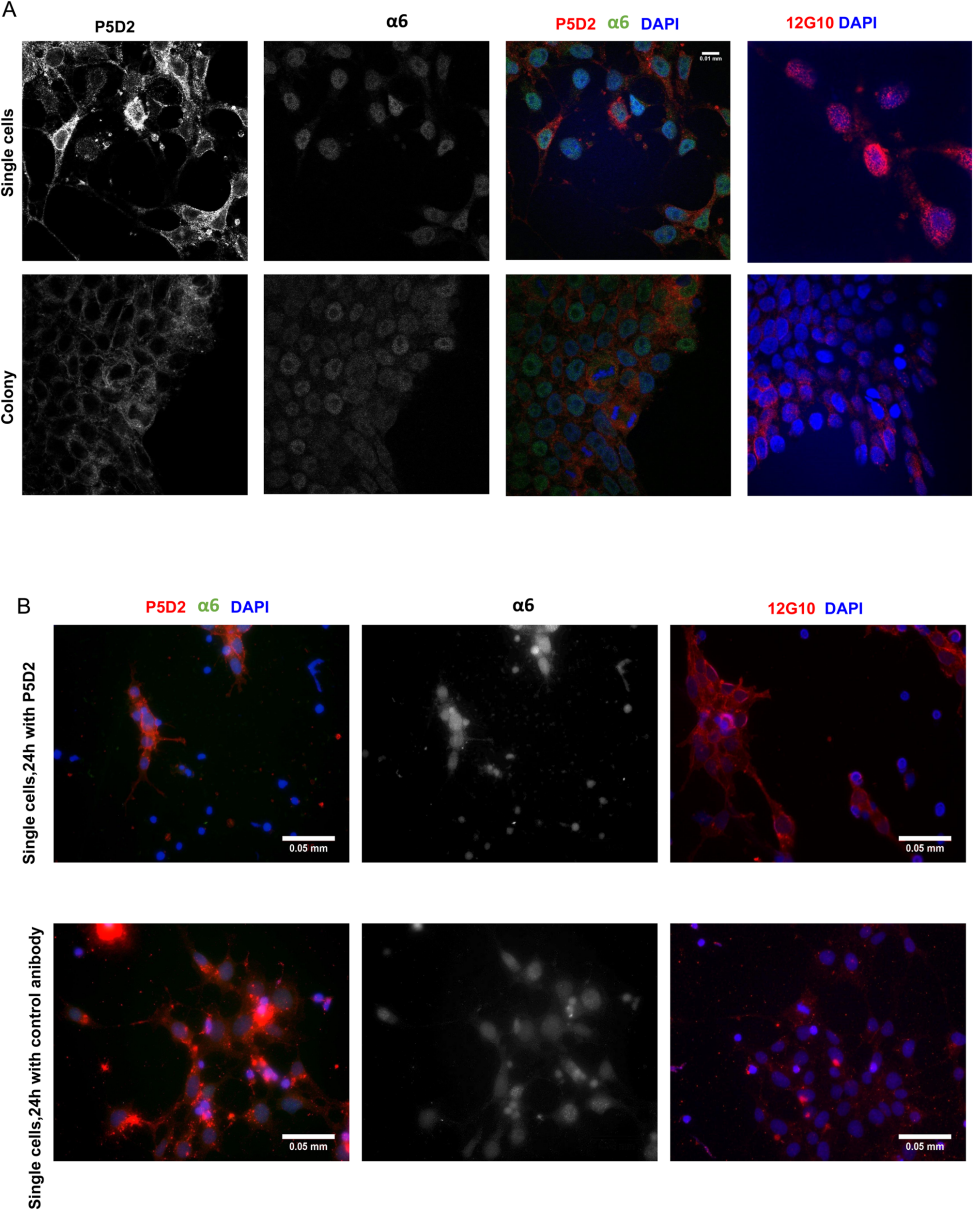
**The localisation of integrins** **β1 and α6 in the hES cells grown in a colony and as a single-cell culture.** hES cells were harvested manually as small colony clumps or detached with EDTA and grown in the presence or absence of Y-27632 (single-cell culture) for 24 h. (A) Integrin β1 was labelled with P5D2 antibodies (recognising total integrin β1) and with 12G10 (associating with active integrin β1) and imaged by confocal microscopy using an oil-immersed objective (60×). Integrin α6 was detected with a respective antibody. (B) The effect of the blocking antibody P5D2 on the localisation of integrin β1. The hES cells were detached with EDTA in the presence of Y-27632 and re-seeded in the presence of the integrin β1-neutralising antibody P5D2 or with an irrelevant control antibody for 24 h. The localisation of integrins β1 and α6 was analysed with fluorescence microscopy using a 40× objective. Scale bars: A, 0.01 mm; B, 0.05 mm.

To assess the localisation of integrin β1 within the cells, we used immunofluorescence microscopy, which showed the formation of integrin β1 clusters within 24 h after re-seeding as a single-cell culture or as a colony (Fig. 2A). In colonies, the highest integrin β1 clustering was detected in the border region. Active integrin β1 had accumulated in dot-like structures, as revealed by 12G10 antibody staining. For detecting integrin α6, we used an antibody, the specificity of which was confirmed by WB (Fig. S1A), but which nevertheless displayed low plasma membrane staining (Fig. 2A). The essentiality of integrin β1 for the adhesion of hES cells to the substrate was confirmed by an experiment in which hES cells were re-seeded onto Matrigel^®^-coated plates in the presence of P5D2, an integrin β1-blocking antibody. The antibody completely abolished the adhesion of small colony clumps (Fig. S2A). However, when the cells were detached with PBS buffer containing 10 mM EDTA into single-cell suspension and re-seeded in the presence of Y-27632 and blocking antibody P5D2, the attachment of some hES cells could be detected (Fig. S2A). On the other hand, the application of an irrelevant antibody as a control method in the same experiment did not cause any changes in the adhesion of hES cells. Since P5D2 abolished the adhesion of manually-detached hES cells entirely, we could analyse how the integrin β1-blocking antibody influenced the levels of integrins β1 and α6 in the single-cell culture. The single-cell culture re-plated in the presence of P5D2 displayed higher integrin β1 staining in some adhered cells which formed cell-cell contacts (Fig. 2B), but the majority of cells had no integrin β1 immunoreactivity. The cells with integrin β1 exposed on the plasma membrane had a very specific morphology with a long leading edge, which was stained with either the antibody P5D2 or the antibody 12G10. Antibody 12G10 stained the functional epitope of integrin β1. In the presence of the irrelevant antibody, which was used in the same experiment instead of P5D2 in the culture medium, cell adhesion remained unaffected and staining the cells with 12G10 showed more dot-like structures. These results, and previous findings that the survival of cryopreserved hES cells can be increased in the presence of Y-27632 (Li et al., 2009), indicate that the inhibition of the Rho-kinase-dependent cell contraction pathway could partially rescue the adhesion and survival of cells, even if the availability of integrin β1 on the cell surface is diminished.

### The internalisation of integrin β1 in hES cells

**Fig. 3.**
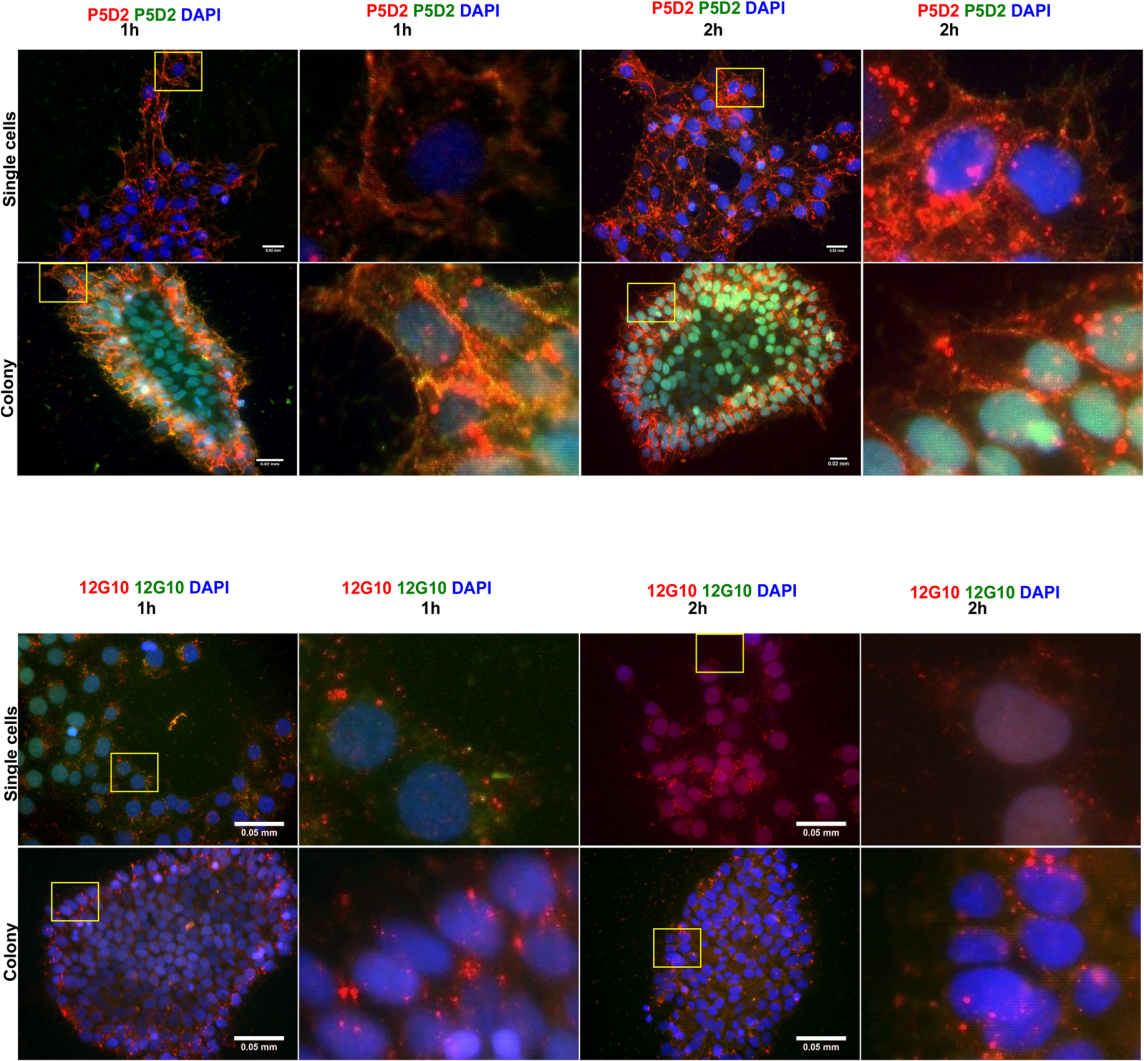
**The internalisation of** **integrin β1 in the hES cells grown in a colony and as a single-cell culture.** The hES cells were incubated with antibodies P5D2 (upper panels) or 12G10 (lower panels) for 30 min on ice and visualised with a secondary antibody conjugated with Alexa Fluor 555 for 30 min on ice. The hES cells were then allowed to grow in an incubator for 1 h or 2 h as described in the Materials and Methods. After that, the hES cells were placed on ice and incubated with another secondary antibody conjugated with Alexa Fluor 488 to visualise the localisation of integrin β1 after 1 h or 2 h of culturing. The boxes are magnified in the following panels. Scale bars: upper panels, 0.02 mm; lower panels, 0.05 mm.

Integrin recycling from the cell interior to the plasma membrane is a dynamic process and essential for cell migration. As shown in carcinoma cells, the internalisation of active and inactive forms of integrin β1 occurs with different efficiencies (Du et al., 2011). In order to characterise the internalisation of integrin β1 in living hES cells, we used the antibodies 12G10 and P5D2. Integrin β1 in hES cells were labelled with antibodies in mTESR1™ medium on ice and hES cells were incubated for 1 or 2 h in a new fresh medium at 37°C (5% CO_2_). After the incubation period, more intensive staining of both active and total integrin β1 was observed in the cells located at the edges of the colony when compared to those at the centre, indicating that the cells in the outer layers internalise integrin β1 more effectively and have the ability to migrate (Fig. 3). The antibody P5D2 marked a distinct area of cells expressing integrin β1 that surrounded the centre of the colony at 1 h and 2 h of incubation. In these cells, integrin β1 (P5D2) was located at the plasma membrane and in the dot-like structures of the cells. After 2 h of incubation, plasma membrane staining caused by P5D2 had diminished and dot-like staining was detectable on the trailing edge the cells located at the colony edges (Fig. 3A). When visualised with antibody 12G10, active integrin β1 localised in dot-like structures as well as in cell protrusions after 1 h. After 2 h of incubation, integrin β1 was visible mostly on the trailing edge of the cells located at the edges of the colony (Fig. 3B). After 2 h, plasma membrane staining had reduced. The dot-like staining localised near the rear of the cells, which could be explained by cell migration. In a single-cell culture, the P5D2 marked the plasma membrane, cell protrusions, and the dot-like structures after 1 h. After 2 h, plasma membrane staining was also detected and the dot-like structures appeared on the trailing edge of the cells. Active integrin β1 localised in dot-like structures after 1 and 2 h. Since active integrin β1 can form focal adhesions, dot-like staining could indicate focal adhesion sites in this experiment, because the living cells were stained with their respective antibodies. As both active and non-active integrin β1 can be endocytosed and recycled within the cells, it might be that some of the dot-like structures were endosomes containing integrin β1. To distinguish between plasma membrane staining and endosomal trafficking of integrin β1 into cells, another antibody was used for detecting integrin β1 after 1 or 2 h of incubation. The other secondary antibody stained integrin β1 on the plasma membrane of hES cells in a single-cell culture and in the cell protrusions of the cells located at the edges of the colony. Some dot-like structures of P5D2 staining were co-stained with another antibody, indicating that these were the focal adhesion sites of the plasma membrane. Still, the larger dot-like structures stained with P5D2 or 12G10 were unreachable for the other antibody, indicating that these dot-like structures were inside the cell. This experiment revealed that subcellular trafficking of integrin β1 in hES cells is substantially different in single-cell cultures, colonies and in different areas of colonies. However, such changes were observed neither in the expression of the pluripotency markers OCT4 and NANOG in different areas of the colony nor in the expression of E-cadherin (Fig. S3).

### Outside-in signalling of integrin β1: RhoA localisation in hES cells and the phosphorylation of myosin light chain

**Fig. 4.**
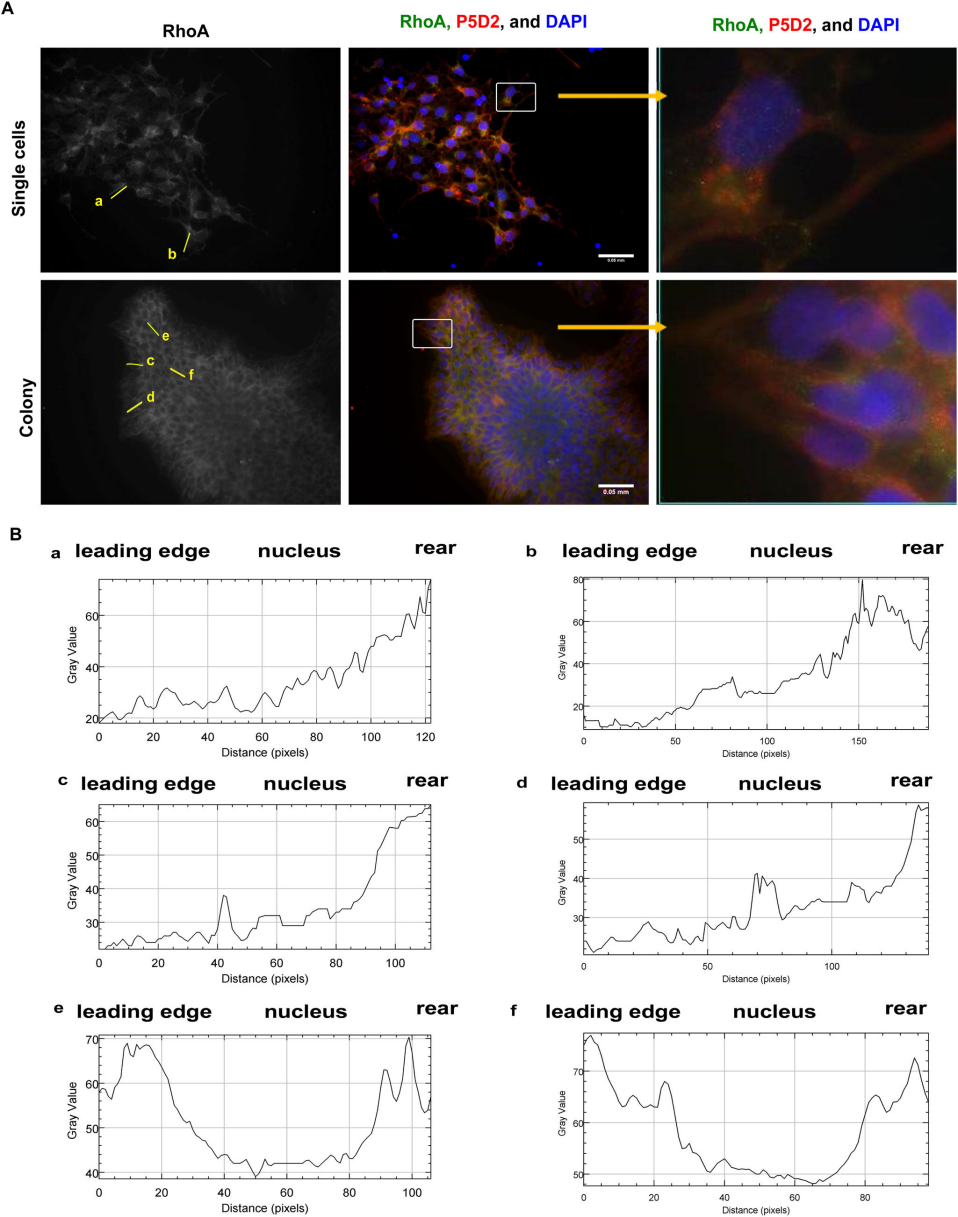
**The co-localisation of RhoA and integrin β1 in the hES cells grown in a colony or as a single-cell culture.** (A) Comparison of immunofluorescence staining of RhoA and integrin β1 (P5D2) in hES cells. The inserts show integrin β1 and RhoA co-staining in a small part of the image and have been presented in next panel. (B) The intensity of RhoA staining in hES cells shown in the first panel was analysed with ImageJ. Fluorescence intensity profiles from a single-cell culture (a,b) and from cells located at the border area (c,d) or at the centre (e,f) of the colony. Scale bar: 0.05 mm.

After having detected differences in integrin β1 trafficking between different areas of the colony and in a single-cell culture, we analysed whether these changes contribute to the signalling pathways regulating the shape of the cells. The shape of a cell depends on the activity of the actin-myosin contraction system, which is mostly regulated by two independent signalling routes: the Ca^2+^-dependent calmodulin/pMLCh system and the Ca^2+^-independent Rho-kinase system. These mechanisms differ in their speed and extent of contraction: the Rho-kinase system provides lower speed and a lesser extent of contraction, whereas calmodulin/pMLCh-powered contraction supports transient and rapid contraction (Katoh et al., 2001). We examined the contribution of both of these regulatory systems to identify possible differences between the cells growing in a colony or as a single-cell culture. To analyse the involvement of the Ca^2+^-independent Rho-kinase system, we examined the co-localisation of RhoA, an upstream regulator of Rho-kinase, with integrin β1 in hES cells. In colon carcinoma cells, the translocation of RhoA from the cytosol to the edges of membrane ruffles, as well as the co-localisation with integrin β1, has been reported to regulate cell migration (O'Connor et al., 2000). In hES cells, the expression of RhoA differed depending on the location of the cell in the colony (Fig. 4A). At the centre of the colony, RhoA was located mostly in the cytosol; at the edges of the colony, however, RhoA staining was observed at the leading edge of membrane protrusions and at a higher level in the cytosol of the trailing edge, as the analysis using the fluorescence intensity profiles of one representative cell indicated (Fig. 4B). Under the conditions existing in a single-cell culture, RhoA co-localised with integrin β1 at the rear of cells and at the leading edge of membrane protrusions (Fig. 4A). In these cells, integrin β1 (P5D2) was confined to dot-like structures, while in migrating cells, it localised in long protrusions, i.e. mostly in the plasma membrane. Thus, the migrating cells had a characteristic RhoA concentration gradient with an increase towards the trailing edge of the cell, whereas in the cells that were at the centre of the colony, RhoA was uniformly distributed within the cell.

To characterise the changes in the Ca^2+^-dependent system, we focused on the phosphorylation of the myosin light chain (MLCh), which triggers the regulation of contractile mechanisms using stress fibres. The myosin light chain is to be phosphorylated at serine 19 and can afterwards interact directly with actin filaments to drive the cell shape changes and contractions (Amano et al., 1996; Getz et al., 2010). In the colonies of hES cells the phosphorylation of myosin light chain was the highest in the cells located at the edges of the colony and formed a circle around the centre of the colony. This border of the colony was highlighted by using an antibody against the cytoskeletal adaptor protein 4.1B (Jung et al., 2011; Wang et al., 2014) which stained the plasma membrane. Cell density as well as the ratio between the areas of the nucleus and the cytoplasm was higher at the centre of the colony. Moreover, these cells were round and without any plasma membrane protrusions. In contrast, the edges of the colony contained cells which were larger and had a higher cytoplasm content than the cells at the centre. To confirm the pluripotency of hES cells in a colony and in a single-cell culture, the expression of NANOG and OCT4 transcription factors was quantified by flow cytometry. A somewhat lower number of cells co-expressing NANOG and OCT4 were observed in a single-cell culture, indicating that these cells are more prone to differentiate. Based on these data, we could conclude that the cells were pluripotent, including the cells at the edges the colony which had different morphology.

**Fig. 5.**
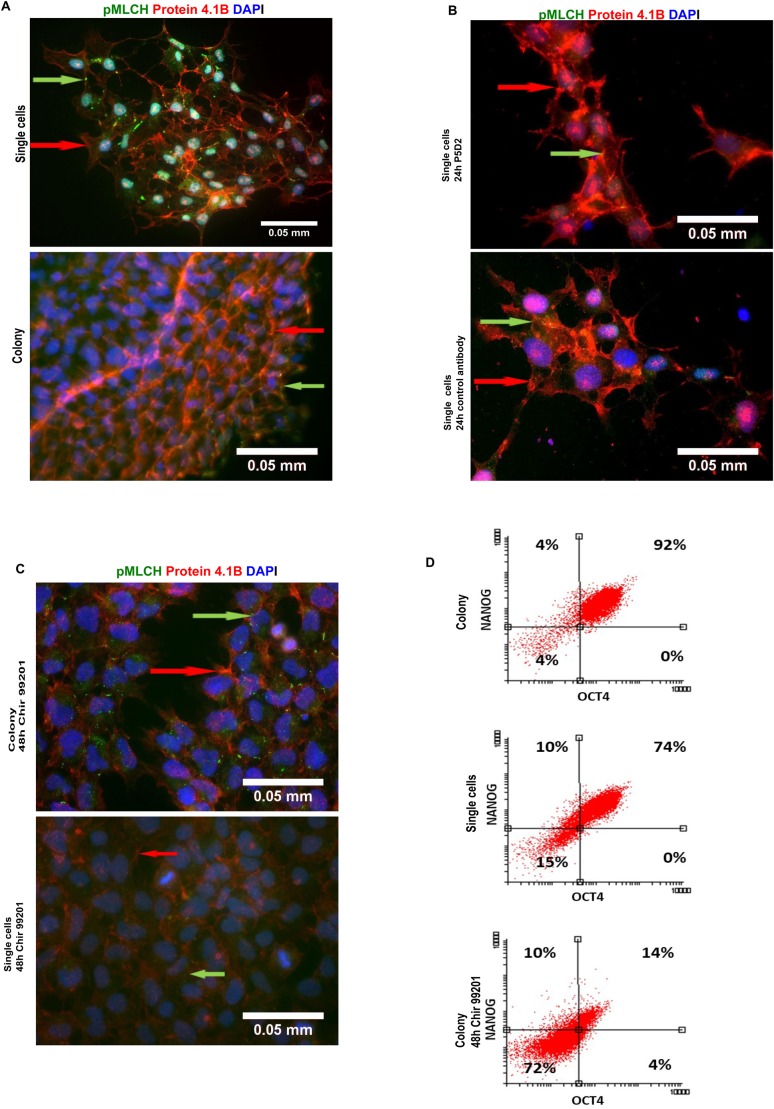
**The comparison of the phosphorylation** **of the myosin light chain (pMLCh) in differently detached hES cells and in the cells differentiated into mesodermal lineage.** The hES cells were grown as described in Fig. 2. For comparing pluripotent (A) and differentiated cells (C), the hES cells were induced to differentiate into mesodermal lineage (induced with CHIR99021 in a differentiation medium). (B) The effect of the blocking antibody P5D2 and the irrelevant control antibody on the phosphorylation of MLCh in hES cells. The plasma membrane of the cells was visualised with an antibody for protein 4.1B and with the phosphorylation of MLCh with a specific antibody detecting phosphorylation at Ser 19. The green arrows indicate the phosphorylation of MLCh and the red arrows point to protein 4.1B staining. (D) The expression of the pluripotency markers NANOG and OCT4 in the hES cells from a colony or from a single-cell culture and in the cells differentiating into mesodermal lineage was quantified by flow cytometry. Scale bar: 0.05 mm.

As expected, the level of pMLCh was lower in a single-cell culture compared to colony and dot-like staining was observed (Fig. 5) in the plasma membranes. As a result of the presence of the integrin β-blocking antibody P5D2 during the re-seeding of hES cells, the number of the dot-like structures of pMLCh decreased in the single-cell culture (Fig. 5). We found protein 4.1B staining on the plasma membrane and dot-like nuclear staining in the single-cell culture. The isoform of protein 4.1B (130 kDa) has been reported to locate primarily in the plasma membrane, while the 60 kDa isoform has been found in the nucleus (Wang et al., 2014). As a result of the presence of the integrin β-blocking antibody P5D2 during the re-seeding of hES cells, the number of the dot-like structures of pMLCh decreased in the single-cell culture (Fig. 5).

In order to compare the pMLCh level in pluripotent hES cells and in differentiated cells, the hES cells were differentiated into mesodermal lineage, which was initiated by re-seeding hES cells after manual detachment or as a single-cell culture. The cultures had no substantial differences in cell density and both cultures showed high expression of the early mesodermal marker brachyury after 48 h and no expression of the pluripotency marker NANOG (Fig. S4), confirming the loss of cell pluripotency. Differentiation into mesodermal lineage significantly suppressed the phosphorylation of MLCh. However, the cells which differentiated from the re-seeded colonies had higher MLCh phosphorylation than the cells differentiated from a single-cell culture (Fig. 5). The level of protein 4.1B in the plasma membrane had diminished in the differentiated cells obtained from both cultures. Moreover, the nuclear staining of protein 4.1B was completely lost, suggesting that this was a characteristic of pluripotent hES cells. Having observed the differences in the phosphorylation of MLCh during cell differentiation, we presumed that it could facilitate further commitment to mesodermal lineage.

### The effect of outside-in signalling of integrin β1 on the localisation of tuberin (TSC2) in hES cells

**Fig. 6.**
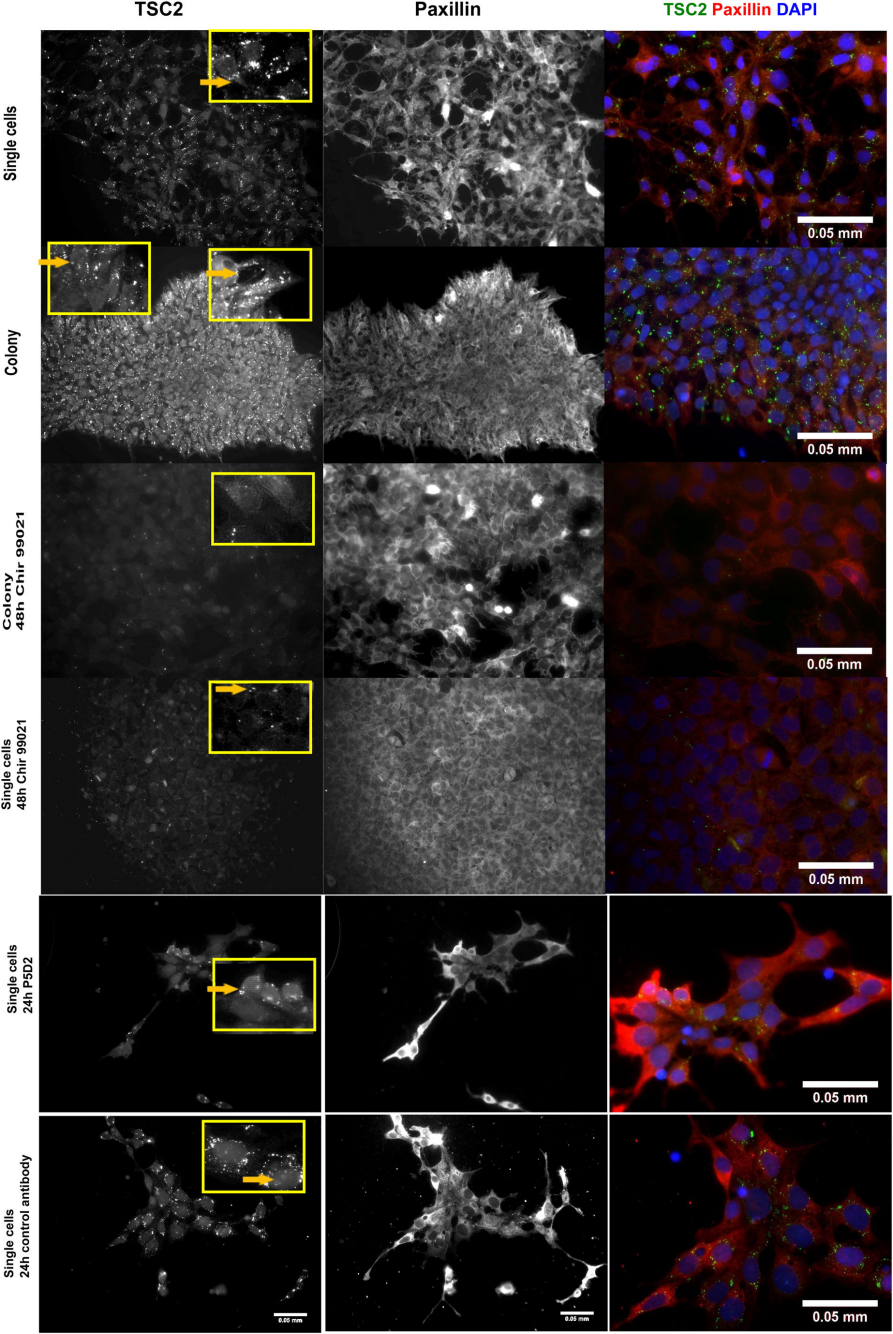
**The localisation of paxillin** **and TSC2 in hES cells and in the cells differentiating into mesodermal lineage.** The cells were stained with TSC2 and paxillin antibodies to visualise focal adhesion sites. The localisation of TSC2 was compared in pluripotent hES cells, in mesodermal progenitors (induced with CHIR99021 in a differentiation medium) and in the hES cells grown in the presence of the antibody P5D2 or an irrelevant antibody as a control method. The yellow boxes, magnified to the right, show the changes in the localisation of TSC2 within the cell. In the image with two yellow boxes, the cells at the centre of the colony can be seen in the left-hand yellow box and the cells at the periphery in the right-hand yellow box. The yellow arrows indicate the localisation of TSC2 in the dot-like structures of the cells. Scale bar: 0.05 mm.

The hES cells located at different areas of the colony could have different access to nutrients and growth factors. The availability of nutrients is sensed by the mammalian target of the rapamycin (mTOR) system and their deficiency triggers the respective signalling pathway. The activation of mTOR signalling is induced by the TSC1–TSC2 (tuberous sclerosis proteins 1 and 2) complex, which is recruited to the lysosome where mTORC1 is located (Demetriades et al., 2016). This allows TSC2 (tuberous sclerosis protein 2) to inhibit mTORC1 by acting on Rheb, which is in part localised on lysosomes. In hES cells, the inhibition of mTOR signalling maintains cells in a pluripotent state, but its activation triggers the expression of developmental genes and subsequent differentiation (Zhou et al., 2009). Thus, we proceeded by analysing tuberin (TSC2) localisation within the cells, since its translocation from lysosomes to the cytoplasm is connected with nutrient status and stress (Rainero et al., 2015). In the colonies of hES cells, TSC2 localised mainly in the small dot-like structures of the cells encircling the colony. However, it localised to the cytoplasm of the cells at the centre of the colony pointing to differences in the activity of mTOR signalling between these cell subpopulations (Fig. 6). In a single-cell culture, TSC2 was found in dot-like structures on the trailing edge of the cells. In the presence of the P5D2 during the re-seeding of the cells, TSC2 localisation in dot-like structures decreased and was almost absent in some cells, suggesting the progress of differentiation processes. However, in the presence of the irrelevant antibody, the localisation of TSC2 in dot-like structures was retained (Fig. 6). During the differentiation into mesodermal progenitors, the dot-like staining of TSC2 was diminished and cytoplasmic localisation was detected.

mTOR signalling regulates the organisation of the cytoskeleton, actin polymerisation and cell morphology (Jacinto et al., 2006). Therefore, the cells were co-stained for paxillin to correlate with the whereabouts of the focal adhesion-associated adapter paxillin and TSC2 lysosomal location. The concentration gradient of paxillin from the leading edge towards the trailing edge could be detected in a single-cell culture. In colonies, an analogous concentration gradient was also characteristic to the cells that displayed the dot-like staining of TSC2 and located in the border of the colony (Fig. 6). The relocation of TSC2 from lysosomes to cytoplasm was found in mesodermal progenitor cells which, unlike hES cells, did not have a paxillin concentration gradient. Thus, the regulation of the mTOR signalling pathway varies in different cells, being dependent on cell-cell contacts and on the position of a particular cell in the hES cell colony.

### The effect of blocking integrin β1 on the formation of embryoid bodies

**Fig. 7.**
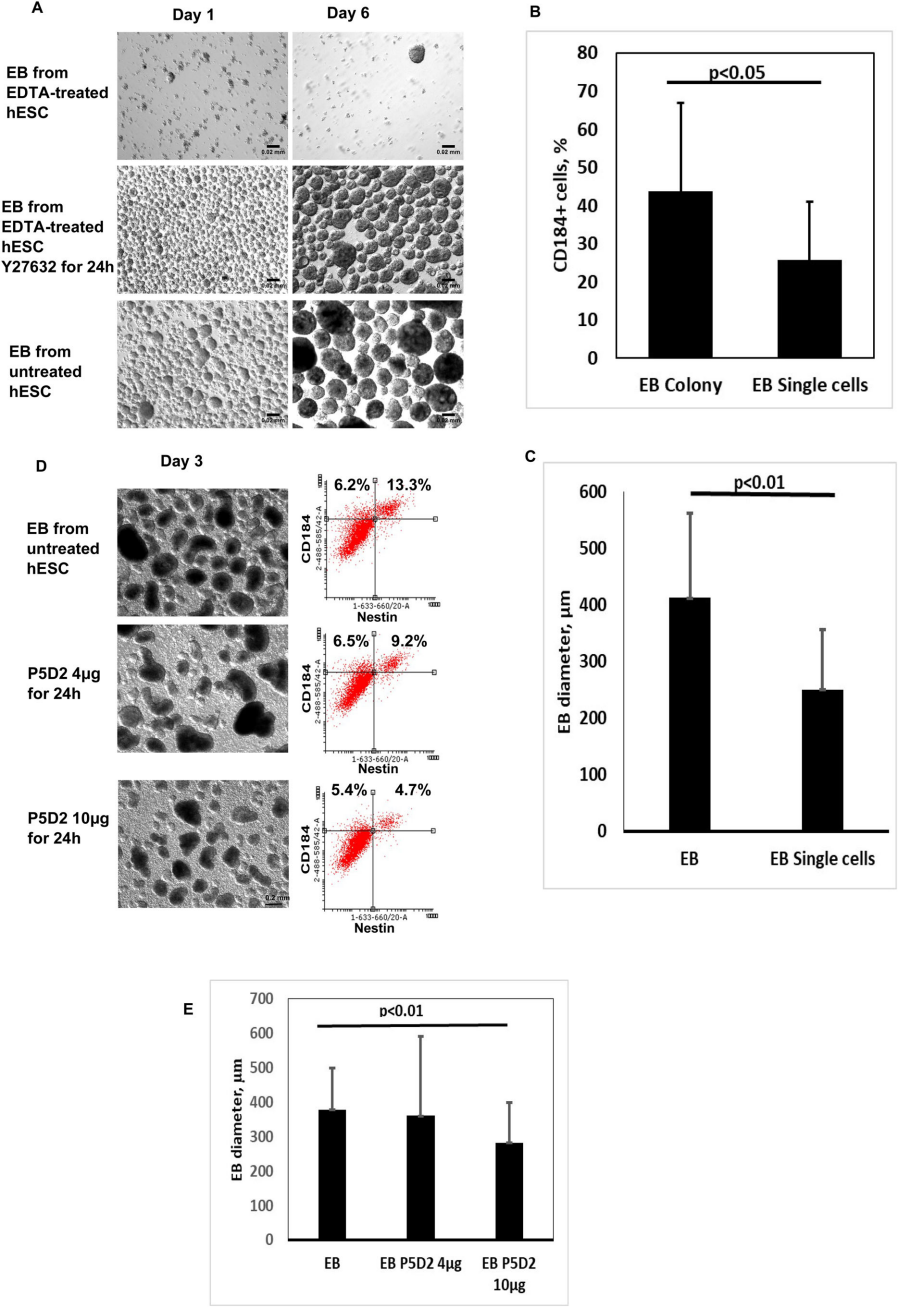
**The effect of integrin β1 on the formation of embryoid bodies (EBs).** (A) Images of EBs formed from colonies or from single hES cells by day 1 and day 6. (B) The number of CD184 (endodermal marker) expressing the cells in the EBs formed from a colony (EB Colony) or from a single-cell culture (EB Single cells) estimated by flow cytometry. (C) Comparison of the size of the EBs formed from a colony and from a single-cell culture analysed based on the images captured with ImageJ at day 6. The data were collected from four independent experiments and are presented as mean±s.d. (D) Concentration-dependent effect of the blocking antibody P5D2 on the formation of the EBs from the colonies of pluripotent hES cells. The next panel shows the dot-plot analysis of the expression of CD184 (endodermal marker) and nestin (ectodermal marker) of the EBs detected by flow cytometry. (E) The size of the EBs formed in the presence of different concentrations of P5D2 (when the EBs were formed from the colonies by manual detachment). Scale bars: 0.02 mm.

Having shown that integrin β1 was crucial for the adhesion of hES cells to Matrigel^®^, we questioned whether integrin β1 is important in the formation of embryoid bodies (EBs). It has been observed that mouse ES cells deficient in integrin β1 led to inadequate deposition of laminin 1 and basement membrane components in the formed EBs (Aumailley et al., 2000). We compared the formation of EBs both in manually and EDTA-detached (in the presence of Y-27632) hES cells. The EBs formed from colony clumps (manual method) were of a different size and lower in number than the EBs formed by using the single-cell culturing method. The morphology of the latter was highly similar to colony-derived EBs, but they were markedly smaller in size (Fig. 7A,C). The EBs formed from single-cell suspension had a lower CD184 expression (endodermal marker, protein and chemokine receptor) than the EBs formed from manually retrieved cells, indicating that the former were in the earlier stage of differentiation than the latter (Fig. 7B). Moreover, the WB analysis showed a decrease in integrin α6 and in the expression of E-cadherin during the differentiation process from pluripotent hES cells to EBs (Fig. S1).

**Fig. 8.**
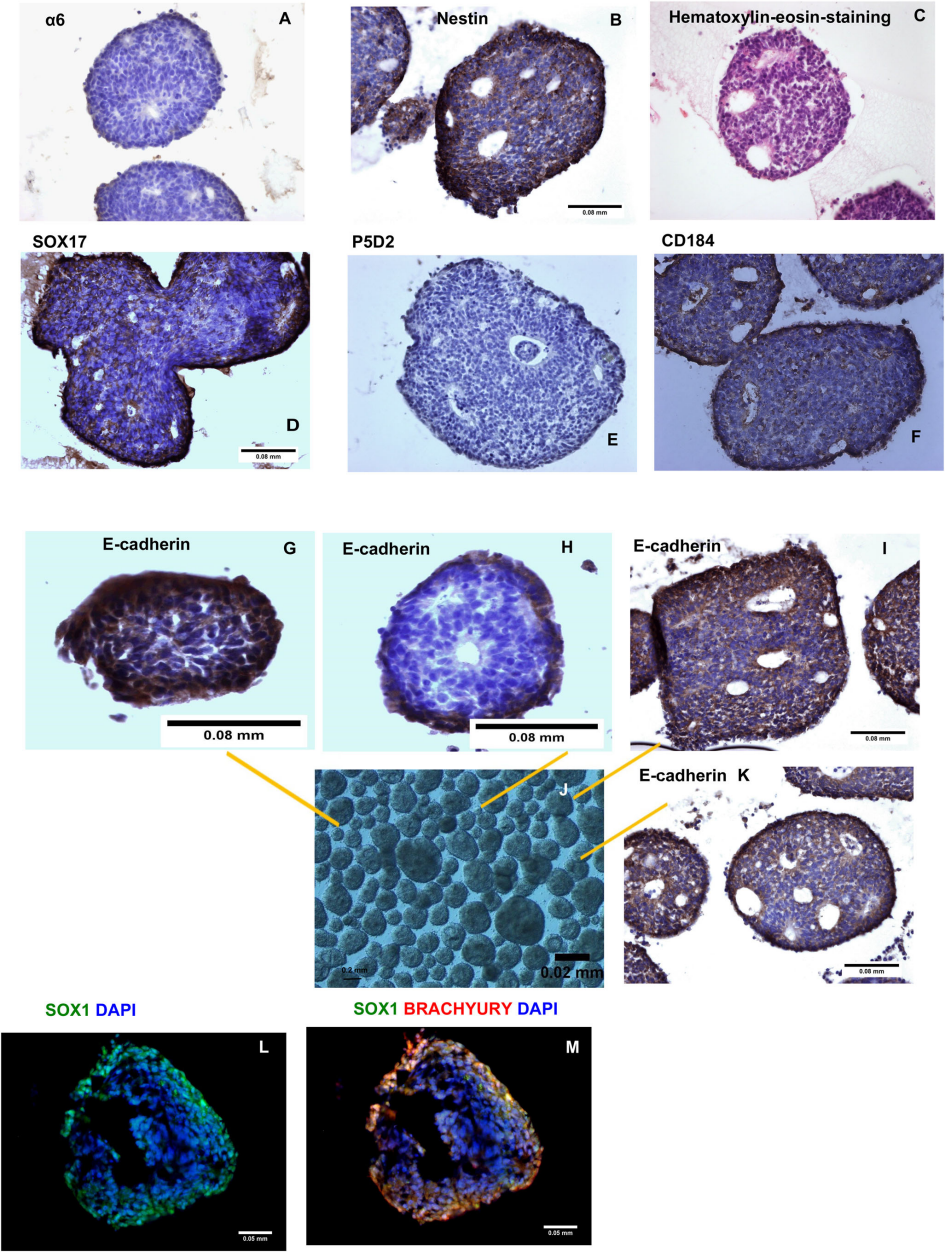
**Immunohistochemical analysis of EBs formed from the colonies of hES cells.** Six-day-old EBs were fixed with 4% PFA and embedded in 3% agarose for further embedding in paraffin. The cross-sections of the EBs were analysed for the expression (brown colour) of integrin α6 (A), integrin β1 (antibody P5D2, E), the ectodermal marker nestin (B) and the endodermal markers SOX17 (D) and CD184 (F). The nuclei were visualised with Haematoxylin and Eosin staining (blue colour). Haematoxylin and Eosin staining shows the morphology of the EBs (C). The localisation of E-cadherin in a small EB (G), in a more differentiated small EB (H), and in a large EB (I,K). EB with various size visualised by light microscope (J). Immunofluorescence analysis of the cross-sections of the EBs for the ectodermal marker SOX1 (L) and co-staining with SOX1 and brachyury (M). The nuclei were visualised with DAPI staining (L,M). Scale bars: A-I,K, 0.08 mm; J, 0.02 mm; L,M, 0.05 mm.

In order to analyse whether the formation of EBs is dependent on integrin β1, the blocking antibody P5D2 was added to the medium for the first 24 h. Upon increasing the concentration of P5D2, the number and size of the formed EBs decreased, indicating that the activity of integrin β1 was impaired; however, it had less influence on the formation of EBs than on the adhesion of pluripotent hES cells to the ECM (Fig. 8). Furthermore, when the cells of the EBs were analysed by flow cytometry, a decrease in the expression level of CD184 and nestin (ectodermal marker) was detected in the EBs formed in the presence of the integrin β1-blocking antibody (Fig. 8), indicating that endodermal and ectodermal lineage differentiation was impaired after blocking integrin β1. When using an irrelevant antibody instead of P5D2, no changes in the formation of EBs were observed (data not shown). These data emphasise the importance of integrin β1 in interfering with the assembly of cell-cell adhesions and the differentiation ability of the cells during the formation of EBs.

For a more detailed characterisation of EBs, we used the immunohistochemical (IHC) method. To overcome the problems of handling the round and small-sized EBs, 6-day-old EBs were first immersed in 3% agarose and embedded in paraffin after gelling. The IHC analysis of the EBs formed from colonies revealed that the expression level of integrin α6 had decreased and moreover it was detectable only in the outer layers of the EBs (Fig. 8A). Furthermore, P5D2 showed no staining of the cells in the EBs acquired with this method (Fig. 8E). The expression of the endodermal markers CD184 (staining in the membrane) and SOX17 (staining in the nucleus) were observed mainly in the outer layers of the cells (Fig. 8D,F), indicating differentiation into endodermal lineage. The ectodermal marker nestin was detectable in the cells surrounding the inner cells of EBs (Fig. 8B), suggesting that there were cells differentiating into ectodermal lineage. The visualisation of E-cadherin revealed the E-cadherin expression gradient within the EBs with an increase towards the outer layers of the cells (Fig. 8G-I). We also found a correlation between the size of the EBs and E-cadherin expression: smaller EBs at earlier stages of differentiation had lower levels of E-cadherin in the cells located at the centre of EBs. When differentiation progressed further and the size of EBs increased, E-cadherin expression was detectable in the outer layers of large-sized EBs and no staining was observed in the inner cells of EBs (Fig. 8G-M). The switch from E-cadherin to N-cadherin during the differentiation process in the monolayers of the cells occurred during epithelial-mesenchymal transition (Eastham et al., 2007). We characterised the cells in EBs further for examining the expression of the mesodermal marker brachyury (Fig. 8M) and ectodermal marker SOX1 (Fig. 8L). Only a few cells were detected to express brachyury (Fig. 8M); SOX1 staining was present mainly in the outer layers of EBs (Fig. 8L,M). Our morphological studies and other reports (Sachlos and Auguste, 2008; Nourse et al., 2010; Esteban-pérez et al., 2016) suggest that the cells in the outer layers of EBs progress more rapidly during the differentiation process than the inner cells of EBs. Therefore, any changes in cell-cell contacts might affect the differentiation potential of the cells in the outer layers. Indeed, yet another report (Brafman et al., 2013) has shown the switch from integrin α6 to integrins α5 and αV during the differentiation into endodermal cells.

## DISCUSSION

The culture of hES cells needs the proper microenvironment for maintaining the pluripotency and differentiation ability. Efficient adhesion of hES cells to ECM components is the first step towards cell survival and growth. This study examined the role of integrin β1 in these processes. We showed that the differences in integrin β1 trafficking in hES cells located both at the centre and the periphery of the colony were also accompanied by different regulation/activity in RhoA and mTOR signalling pathways. In this study, we revealed that cell morphology, as well as the contraction, migration and differentiation ability, depend on the position of a particular cell within the colony.

The evaluation of integrin β1 activity and regulation in hES cells is a complicated task since active conformation is needed for functional activity, but both the active and inactive form is recycled within the cells to regulate cell adhesion and migration. The methods used in this study for analysing the levels of integrin β1 on the plasma membrane of living cells provided information about its availability for interacting with the components of ECM. This approach enabled us to measure the level of integrin β1 on the plasma membrane and its association with functional activity. Furthermore, the differences in the signalling activity of integrin β1 were found to correlate with dissimilar regulation of RhoA and mTOR signalling pathways. Remarkably, a dissimilar activity of these signalling pathways allowed us to distinguish between two distinct sub-populations in the colonies of hES cells – the peripheral and central cells of a colony. These findings could explain the inherent heterogeneity of hES cells grown in a colony, which has so far been poorly understood, especially considering the fact that no significant differences have been discovered in the expression of pluripotency markers so far.

Currently, hES cells are mostly re-seeded as smaller fragments of a colony or as a single-cell suspension obtained from EDTA-induced dissociation and grown further in the presence of the ROCK-inhibitor Y-27632. Comparing these two different re-seeding methods allowed us to demonstrate that the hES cells grown as a single-cell culture showed more efficient integrin internalisation and RhoA concentration gradients. Remarkably, similar properties were also possessed by the hES cells located at the periphery of a colony. A distinct area of cells surrounding the centre of a colony was bordered with an actin-myosin contraction network that included the structural protein 4.1B. A circular actin-myosin structure that surrounds the core of a colony has also been reported previously (Närvä et al., 2017) and our results support this finding. Another study (Nakashima and Omasa, 2016) showed that differences exist in the force between the cells at the centre of the colony and at the periphery without any substantial differences in the expression of pluripotency markers. The same study reported that the cells populating the edges of the colony started to differentiate into endodermal lineage earlier than the cells at the centre (Nakashima and Omasa, 2016). Together with our results, these findings provide useful information about the colony structure, morphology, and signalling network of hES cells, which could also contribute to a better understanding of the colony formation by other cells, such as tumours.

The hES cells grown as a single-cell culture are more homogenous than the cells grown in a colony. These cells were found to also have higher migration potential and lower MLCh phosphorylation levels compared to the cells grown in a colony. Furthermore, a somewhat lower expression of the pluripotency factors OCT4 and NANOG was detected, indicating that a single-cell culture was more prone to differentiate. Indeed, the EBs formed from a single-cell culture were more similar in size, suggesting that a single-cell culture has higher differentiation potential. A single-cell culture could therefore be preferred in differentiation protocols to decrease the heterogeneity and increase the yield of differentiated cells.

When RhoA-kinase activity was inhibited under single-cell culturing conditions with Y-27632, the cells were able to migrate, form colonies, and differentiate into EBs. In contract, in the hES cells grown in a colony the differences depended on the location of the cells: at the centre, the RhoA-kinase controlled actin-myosin contraction was highly activated, while at the periphery the cells had a RhoA concentration gradient, were more prone to migrate and had higher internalisation of integrin β1. In highly metastatic cells, the use of Y-27632 has shown to be effective in decreasing the migration, while in non-metastatic rat embryonic fibroblasts it increases invasiveness (Wang et al., 2016; Rosel et al., 2008). Together with our findings, it confirms a link between the RhoA-kinase system, the availability of integrin β1 for the adhesion of cells on the plasma membrane and the pluripotency of cells, as a somewhat lower number of cells co-expressing NANOG and OCT4 was detected in a single-cell culture than in the cells from a colony.

To characterise the involvement of the mTOR signalling network, we studied the localisation of TSC2, which was found mainly in the dot-like structures of pluripotent hES cells. The localisation of TSC2 in lysosomes, resulting in the inactivation of mTOR signalling has been reported in connection with starvation conditions or in response to multiple individual stresses (Rainero et al., 2015). It could be that mTOR signalling was suppressed by the microenvironment used for maintaining hES cell cultures in their pluripotent state. However, during the differentiation into mesodermal lineage, the conditions were probably less stressful and resulted in the translocation of TSC2 into a cytoplasm, since we noticed a decreased number of dot-like structures with TSC2 staining. The reason behind analysing the differentiation into mesodermal lineage is that the induction of mesodermal progenitors is utilised as the first step in differentiating into endothelial cells which express high levels of integrin α6β1 (Lian et al., 2014; Sriram et al., 2015; Caiado and Dias, 2012; Toya et al., 2015). In mouse ES cells, the interaction between integrin α6β1 and laminin 1 has been demonstrated to determine the efficacy of the differentiation into endothelial cells (Toya et al., 2015). Despite several differentiation protocols, the high heterogeneity of endothelial cell progenitors has been one of the obstacles to overcome. This study evaluated the role of integrin β1 in pluripotent cells as well as during the early stages of differentiation to observe the differences between the hES cells grown in a colony or as a single-cell culture.

The functional activity of integrin β1 has dissimilar roles in pluripotent hES cells and in differentiating cells forming EBs. In the presence of the integrin β1-blocking antibody (P5D2), the formation of EBs was impaired, whereas the same antibody completely abolished the adhesion of the pluripotent hES cells to Matrigel^®^ and eventually led to cell death. When analysing 6-day-old EBs using IHC analysis, a novel method that we introduced for the characterisation of EBs, integrin β1 with its antibody P5D2 was not detected in the EBs. The WB method, however, confirmed that the integrin β1 protein was still present in EBs. The downregulation of integrins β1 and α6 in the cells differentiating into endodermal lineage for monolayer cell cultures has been reported (Brafman et al., 2013). Therefore, it was not surprising that these EBs had no staining for integrins β1 and α6. The expression pattern of integrins (as well as other adhesion molecules) changes at different time points during the formation of EBs, being more complex than currently known. During the first 24 h of EB formation, blocking integrin β1 impaired the formation of EBs by decreasing the size and number of EBs as well as the differentiation potential of the cells in the outer layers, but upon further progression of EB growth, integrin β1 became less important. We could characterise the generation of the E-cadherin concentration gradient during the progression of differentiation. It was interesting to observe that the differentiation processes were most rapid in the cells in the outer layers of EBs. Furthermore, the expression of endodermal and ectodermal markers was detectable in these cell layers. Since we tested 6-day-old EBs, the visceral endoderm and ectoderm could not be completely distinguished from one another. Still, the information obtained from the IHC analysis offered new insight into the commitment process of cells within EBs and emphasised that the differentiation process is highly regulated and depends on the localisation of cells. Thus, the IHC method is a useful tool for studying highly dynamic differentiation processes in EBs. The adhesion of EBs warrants further investigation, especially regarding the involvement of integrins in this process.

## CONCLUSION

The availability of integrin β1 on the plasma membrane of hES cells is required for the adhesion of cells to the ECM. Attachment is a crucial step for the survival of hES cells and is necessary for maintaining the pluripotency and differentiation potential required for forming EBs. Various protocols have been utilised to differentiate hES cells in order to produce specialised cells in quantities required for translational therapy. This study shows the high impact of integrin β1 in mediating the adhesion of hES cells to the ECM, which is essential for the survival of the cells as well as for the formation of EBs. Thus, the integrin-mediated outside-in signalling could also influence the success and yield of the differentiation procedures of hES cells.

## MATERIALS AND METHODS

### Ethics statement

This study was conducted using a commercially available human embryonic stem cell line (WA09 - H9, National Stem Cell Bank, Madison, USA). No *in vivo* experiments using animals or human subjects were performed, and therefore, approval from an ethics committee was unnecessary.

### Cell culture

H9 ES cell line (WA09, National Stem Cell Bank, Madison, USA) was maintained on Matrigel^®^- (BD Biosciences, San Jose, USA) coated plates in a mTeSR1™ maintenance medium (STEMCELL Technologies Inc., Vancouver, Canada) in accordance with the manufacturer's specifications. The medium was replaced on a daily basis. After 3–4 days of growth, the colonies were detached mechanically using a micropipette tip (manual scraping technique). After breaking up the colonies into smaller parts with gentle pipetting, the hES cell clumps were plated onto separate new Matrigel^®^-coated plates.

The normal karyotype of cells was confirmed by using G-banding.

### Antibodies and reagents

The following primary antibodies were used: 12G10 (anti-active β1 integrin), P5D2 (anti-β1 integrin, blocking antibody), anti-E-cadherin, anti-protein 4.1B (all from Santa Cruz Biotechnology), anti-α6 integrin antibody (LSB Biotech), anti-TSC2, anti-RhoA, anti-phosphorylated myosin light chain (all from Cell Signaling Technology), anti-SOX17 and anti-beta-actin (both from Abcam). The secondary antibodies were used as shown in Table S1. Anti-NANOG, anti-CD184 (PE conjugate), anti-nestin (Alexa-647 conjugate) antibodies and their isotype control antibodies were purchased from BD Biosciences. Anti-brachyury and anti-SOX1 antibodies were purchased from R&D Systems (Abingdon, UK). The reagent used in mesodermal lineage differentiation (CHIR99021) was purchased from Sigma-Aldrich Chemicals.

### Immunofluorescent analysis

The hES cells were harvested either manually or with EDTA (10 mM in PBS, 3 min) and re-seeded to new Matrigel^®^-coated four-well plates with the mTeSR™^1^ medium in the presence or absence of Y-27632 (10 µM). After 24 h, the cells were fixed using a two-step fixation method. First, 4% paraformaldehyde (PFA) solution in PBS (fixing solution) was added to the medium (ratio 1:5) and incubated for 2 min. After aspiration, the cells were fixed with the fixing solution for 10 min at room temperature (RT). Fixed cells were stored in PBS at 4°C. For detecting intracellular antigens, hES cells were permeabilised with a permeabilisation buffer (permeabilisation buffer, e-Biosciences) for 20 min at RT, then blocked with 2% normal goat serum (NGS; PAA Laboratories, Linz, Austria) for 30 min and incubated with primary antibodies for 1 h at RT. hES cells were washed four times for 3 min with TBS containing 0.1% Tween 20. The secondary antibodies were used as shown in Table S1. The cells were incubated with secondary antibodies for 1 h at RT in the dark. DAPI (Sigma-Aldrich) was used as a nuclear counterstain. The samples were mounted with Fluorescent Mounting Medium (DAKO) for further imaging using a fluorescence microscope (Olympus BX51) with Cell^B image-acquisition software (Olympus). Confocal microscopy was performed with the Olympus IX81 inverted microscope equipped with the FluoView FV1000 confocal laser scanning system (Olympus, UK). Images were processed and analysed using the ImageJ software.

### Flow cytometry

For detection of integrins β1 and α6 on the surface of hES cells, the cells were either harvested manually with EDTA (10 mM, 3 min) or with 0.05% trypsin-EDTA solution (PAA Laboratories, Linz, Austria) for 5 min and afterwards washed with PBS containing 2% fetal bovine serum (FBS). The single cells were suspended in 100 µl PBS containing 1% of BSA, and 2 mM EDTA on a 96-well low-adsorption microplate and the plate, which was lifted on ice. The cells were blocked using 2% NGS in PBS containing 1% of BSA and 2 mM EDTA (10 min), and stained for 30 min on ice with the appropriate antibodies for detecting integrins β1 and α6 or their isotype control antibodies. After washing with PBS (1% BSA, 2 mM EDTA), the cells were incubated with goat anti-mouse Alexa Fluor 647 or chicken anti-rabbit Alexa Fluor 488 antibodies. Flow cytometry data were acquired with FACSAria using FACSDiva software (BD Biosciences). The populations that were positive or negative for specific markers were selected using density plots. The borders of the populations were defined by using specific biological samples (trypsin-treated hES cells) and were also confirmed with specific isotype controls.

In analysing the differentiation markers of the cells from the EBs, the EBs were dissociated into single cells by extensive pipetting and fixed using 1.6% paraformaldehyde (PFA; Sigma-Aldrich) solution for 10 min at RT. The cells were washed and stained using the permeabilisation buffer, blocked with 2% NGS in the permeabilisation buffer (10 min) and stained with the appropriate antibodies or their isotype control antibodies for 30 min at RT. For cell cycle analysis, the cells were stained with DAPI (Sigma-Aldrich).

### Antibody-based integrin β1 internalisation and recycling assay

The hES cells were cultured for 24 h after re-seeding after which four-well plates were placed on ice and allowed to cool down. Integrin β1 on the cell surface was labelled with antibodies P5D2 or 12G10 (1:1000) for 30 min on ice, washed with medium (mTeSR1™) three times and incubated with goat anti-mouse Alexa Fluor 555 for 30 min on ice in the dark. After washing with a medium, the medium was replaced with a new one and the plates were incubated in the incubator at 37°C for 1 h or 2 h (CO_2_ 5%). At the indicated time, the plate was placed on ice and the cells were labelled with donkey anti-mouse Alexa Fluor 488 (1:1000) for 30 min on ice. After washing with PBS (without Ca^2+^, Mg^2+^), the cells were fixed as described above, then stained with DAPI, and mounted with Fluorescent Mounting Medium for further imaging using a fluorescence microscope.

### Differentiation of hES cells into embryoid bodies

For the formation of EB, the suspension method was used: hES cells were either manually scraped or dissociated with 10 mM EDTA-PBS for 3 min from the Matrigel^®^ plates and then 3D embryonic bodies were let to form in Essential 6 Medium (Thermo Fisher Scientific) on a low attachment culture plate in the presence or absence of 10 µM Y-27632. The medium was replaced with a new medium (without Y-27632) after 2 days. The formation of EBs was assessed visually using a microscope with a 37°C heated stage. Formed EBs were collected on day 6 for histological assay, IHC analysis, flow cytometry and WB analysis.

### Differentiation of hES cells into mesodermal lineage

The hES cells were dissociated and detached from Matrigel^®^-coated plates either manually or with 10 mM EDTA in PBS for 3 min and re-seeded onto a new Matrigel^®^-coated plate in the presence or absence of 10 µM Y-27632 in the mTeSR1 medium for a further 24 h. After that, the medium was changed to Essential 6 Medium containing 5 µM CHIR 99021 and cultured for 48 h without changing the medium. The expression of the differentiation markers was assessed on day 2.

### Histological assays

The 6-day-old EBs were fixed with 4% PFA in PBS for 30 min at RT, washed with PBS, embedded in 3% agarose gel, and processed for paraffin embedding by using standard methods (Buesa, 2007). Serial sections (∼8 µm) were prepared using Microm HM355S (Microm International GmbH, Walldorf, Germany). Deparaffinised sections were stained using Haematoxylin and Eosin (Buesa, 2007). The images of cross-sections were analysed using Cell^B image acquisition software.

### Immunohistochemistry

In the deparaffinised sections of the EBs, the localisation of analysed proteins was revealed using the following antibodies: mouse anti-Sox17, mouse anti-CD184, mouse anti-integrin β1, rabbit anti-integrin α6 and mouse anti-E-cadherin. The sections were processed with Mouse and Rabbit Specific HRP/DAB (ABC) Detection IHC kit (ab64264; Abcam) in accordance with the manufacturer's protocol. The sections were embedded into Histomount (Life Technologies). The images of cross-sections were analysed using Cell^B image acquisition software.

### Statistical analysis

A one-tailed paired *t*-test with a confidence interval of 95% was performed with GraphPad Prism 4 software (GraphPad, San Diego, USA). The results have been presented as the mean±of the standard deviation.

## Supplementary Material

Supplementary information
