# Supplementary material for: The role of integrin β1 in the heterogeneity of human embryonic stem cells culture
Source: Biol Open. 2018 Nov 15;7(11):bio034355. doi: 10.1242/bio.034355 (PMC6262870; doi:10.1242/bio.034355)
Supplement: Supplementary information [file biolopen-7-034355-s1.pdf]

## Supplementary Methods

*Western blot (WB) analysis*

The hES cells and embryoid bodies (EBs) were lysed in the RIPA buffer containing 10 mM Tris-HCl (pH 7.5), 150 mM NaCl, 0.1% SDS, 0.3% Triton X-100, 0.3% sodium deoxycholate, 2 mM EDTA, and the Complete protease inhibitor cocktail (Roche Diagnostics) and stored at -20 ˚C. The concentration of the proteins in the samples was measured with the BCA Protein Assay Kit (Thermo Fisher Scientific) and equal amounts of protein were electrophoresed in an 8% SDS polyacrylamide gel and transblotted with the Mini Trans-blot Cell system (Bio-Rad, Hercules, CA) onto a polyvinylidene difluoride membrane (Millipore, Billerica, MA). The membranes were blocked with a 5% (w/v) non-fat dry milk powder (AppliChem) solution in PBS containing 0.1% Tween-20 (blocking solution). The incubation with primary antibodies was performed in the same blocking solution overnight at 4 ˚C, which was followed by an incubation with a secondary antibody in a blocking solution for 1h at RT. The membranes were probed with rabbit anti-integrin α6 antibodies, mouse anti-integrin β1 antibodies (Santa Cruz Biotechnology), and mouse anti-E-cadherin antibodies (Abcam, Cambridge, MA, USA), which was followed by the inclusion of the horseradish peroxidase-conjugated goat anti-rabbit or goat anti-mouse secondary antibodies (Cell Signaling). The mouse anti-β-actin antibody (Abcam) was used for detecting the loading control. The binding of the antibodies was detected with the Immobilon Western Chemiluminescent HRP Substrate solution (Millipore Corporation). The membranes were exposed to the x-ray films (Agfa, Belgium) used for the chemiluminescent signal detection. Plots were analysed using the ImageJ gel plot analysing tool.

Biology Open • Supplementary information

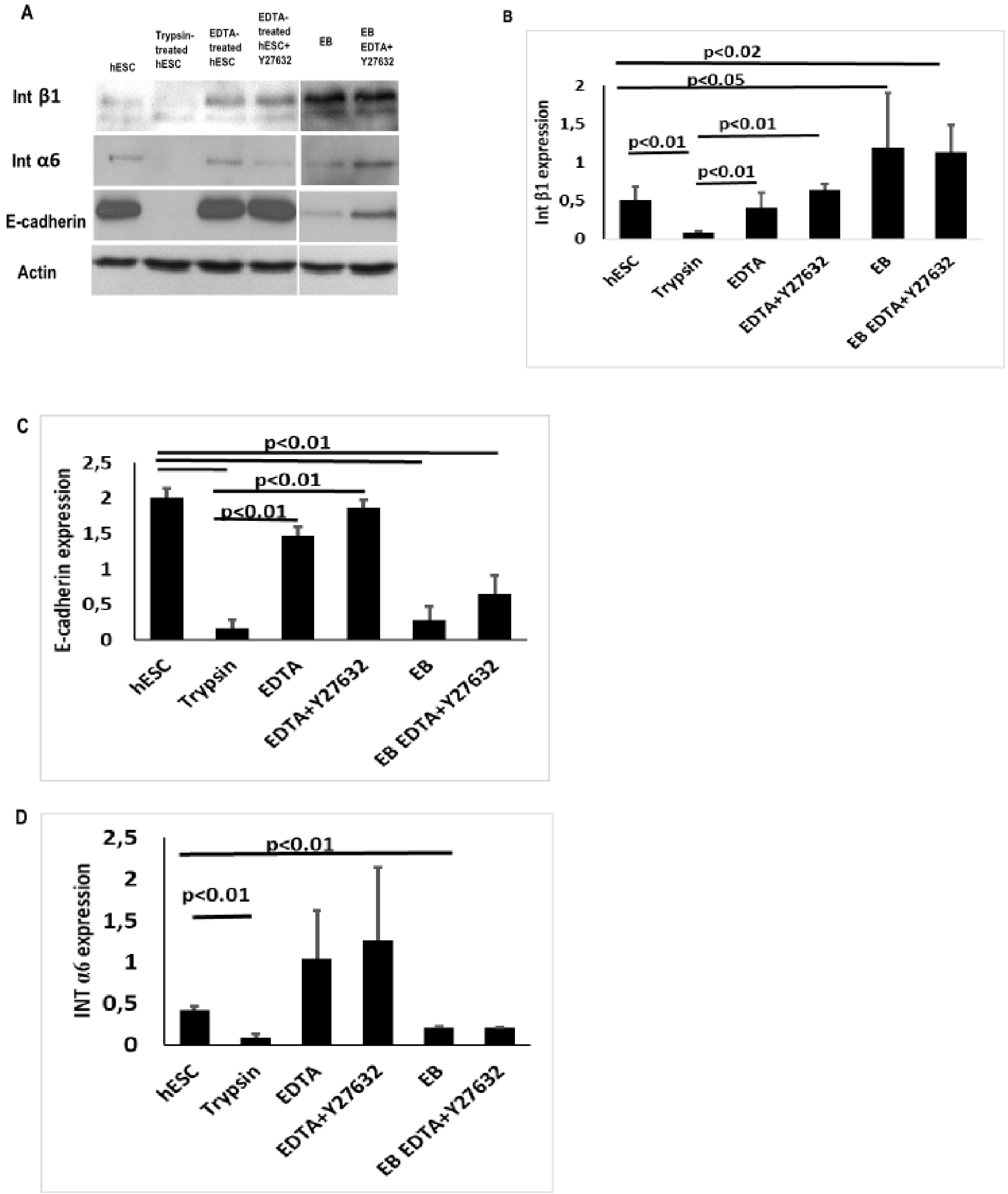


**Fig. S1. The levels of integrins β1 and α6 as well as E-cadherin of the hES cells detached with different methods measured by Western blot/WB analysis.**

(A) The WB analysis of the hES cells detached with different methods and the embryoid bodies (EB) formed from the hES cells detached manually or with EDTA and in the presence of the Rock inhibitor.

Biology Open • Supplementary information

(B) Relative quantification of integrin β1 levels to β-actin levels using the ImageJ gel plot analysing tool. The data have been collected from three independent experiments and are shown as mean ± SD. $P < 0.01$ is considered to be statistically significant.

(C) Relative quantification of integrin α6 level to β-actin level using Image J gel plot analysing tool. The data have been collected from three independent experiments and are shown as mean ± SD. $P < 0.01$ is considered to be statistically significant.

(D) Relative quantification of E-cadherin level to β-actin level using Image J gel plot analysing tool. The data have been collected from three independent experiments and shown as mean ± SD. $P < 0.01$ is considered to be statistically significant.

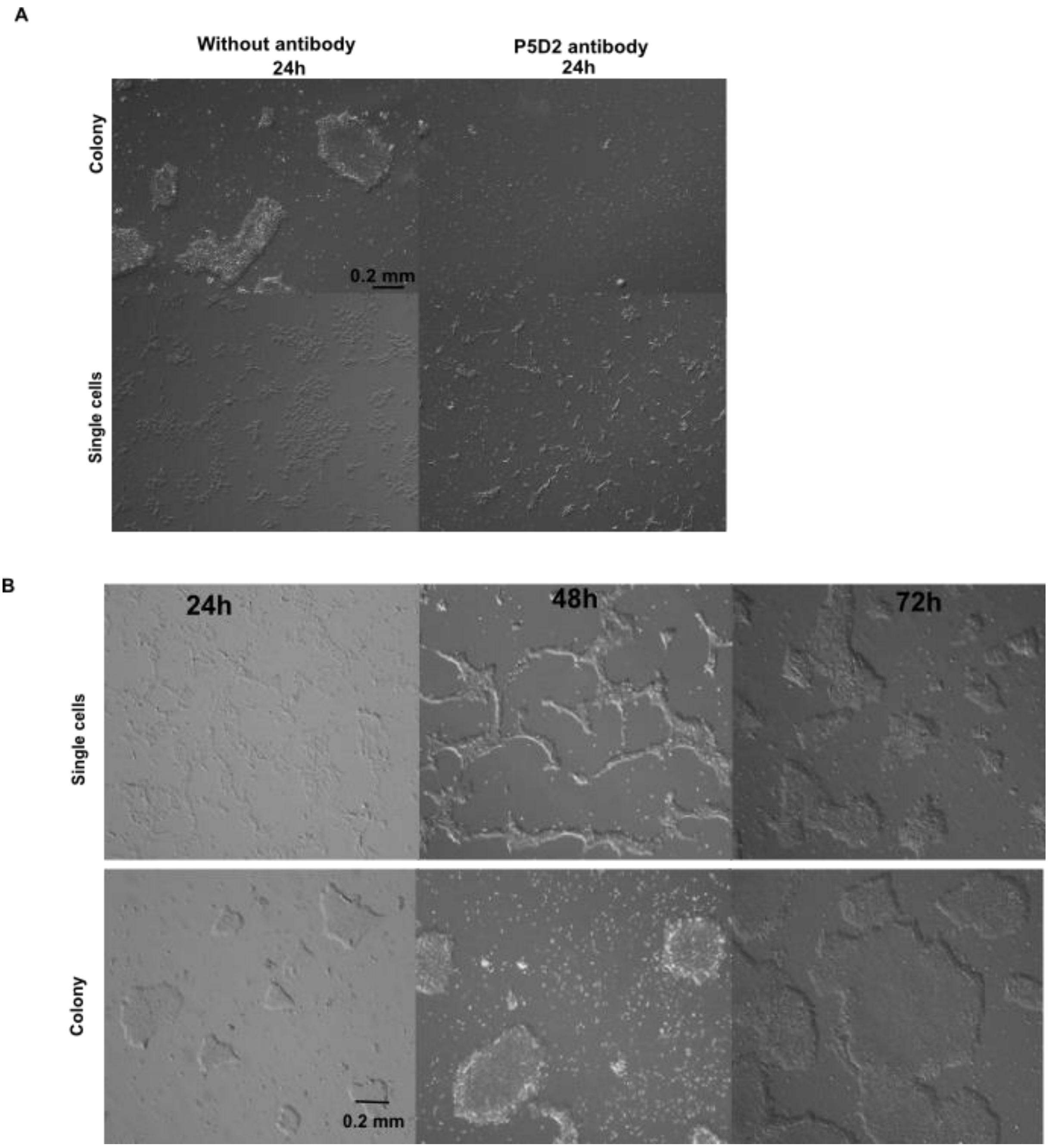


**Fig. S2. The formation of the colonies of hES cells and growth in a single-cell culture.**

(A) The effect of the integrin β1-blocking antibody P5D2 on the attachment of hES cells after having been detached manually or with EDTA in the presence of Y-27632 and re-seeded to the Matrigel®-coated 6-well plate.

(B) The effect of different detachment methods on the growth of hES cells during 72h.

Biology Open • Supplementary information

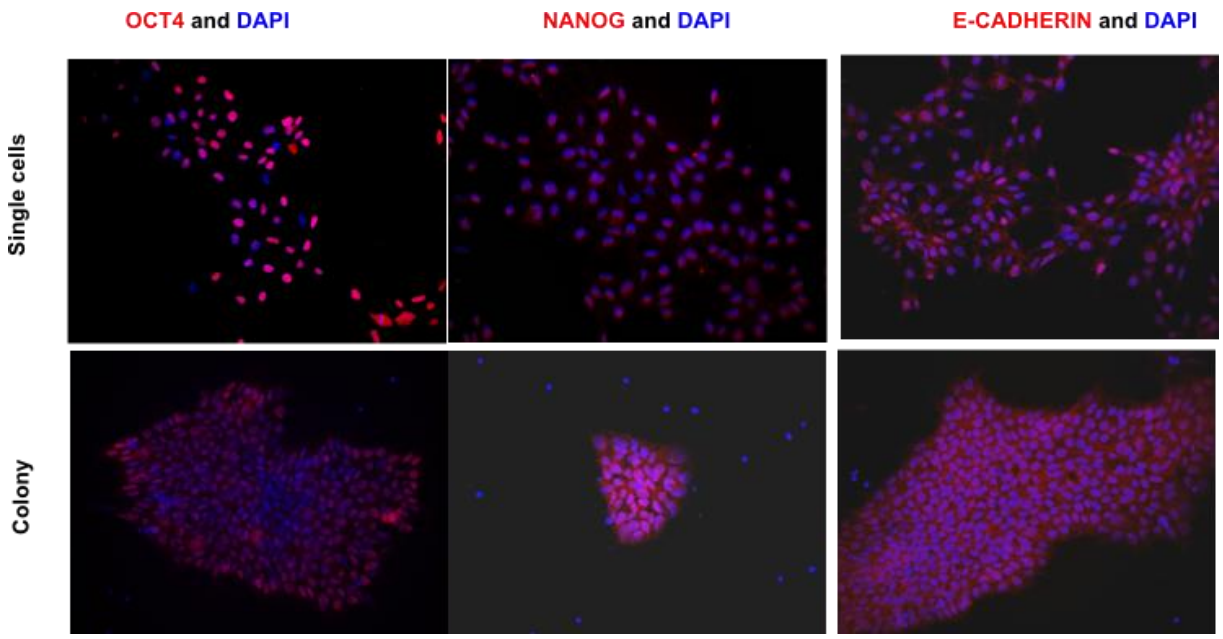


**Fig. S3. Characterisation of pluripotency markers in hES cells in single-cell culture and in colony.** Immunofluorescence staining of transcription factors NANOG, OCT4, and E-cadherin in hES cells 24h after re-seeding.

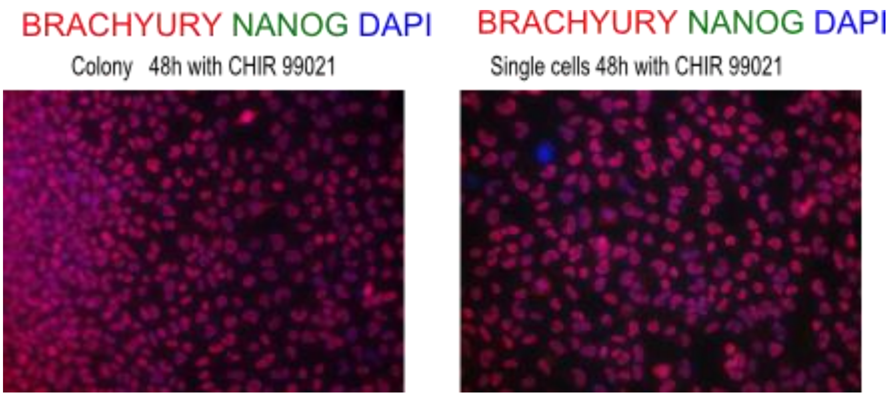


**Fig. S4. Differentiation of hES cells into mesodermal lineage.** The expression of the mesodermal marker BRACHYURY and the pluripotency marker NANOG in the cells differentiated with CHIR99201 in the Essential 6 medium for 48h. The hES cells were detached manually or grown as a single-cell culture for 24h before differentiation was initiated.

**Table S1.** Antibody clones used for different assays.

| Target | Clone | Manufacturer | Assay |
|---|---|---|---|
| Integrin β1 | P5D2 | SC-13590 | IF, neutralisation assay, internalisation assay |
| Integrin β1 | 12G10 | SC- 59827 | IF, internalisation assay |
| Integrin β1 | B3B11 | Ab77803 | WB, FACS |
| Integrin α6 | | LSC 38650 | IF, WB |
| Protein 4.1B | B-6 | SC-398089 | IF |
| RhoA | 67B9 | 2117S Cell Signaling Technology | IF |
| TSC2 | | 3612S Cell Signaling Technology | IF |
| Phosphorylated Myosin light chain 2 (T18/S19) | | 3674S Cell Signaling Technology | IF |
| Paxillin-TRITC (1-557) | | 610055 BD | IF |
| E-cadherin | 3-10 | SC-8426 | IF, FACS |
| CD184 | | SC-53534 | FACS |
| Nestin Alexa Fluor 647 | | 560341 BD (lot 4346985) | FACS, IHC |
| CD184 PE | | 557145 BD | FACS, IHC |
| Nanog | N31-355 | 560483 BD | IF |
| OCT4 | C-10 | SC-5279 | IF |

Secondary antibodies

| | Reference number | Manufacturer |
|---|---|---|
| Chicken anti-rabbit Alexa Fluor 488 | A21441 | Life Technologies |
| Goat anti-mouse Alexa Fluor 647 | A21463 | Invitrogen |
| Goat anti-mouse Alexa Fluor 555 | A21424 | Invitrogen |
| Donkey anti-rabbit Alexa Fluor 488 | A21207 | Invitrogen |
| Donkey anti-mouse Alexa Fluor 488 | A210202 | Invitrogen |
| Donkey anti-goat Alexa Fluor 594 | 11058 | Invitrogen |

Biology Open • Supplementary information
